# New Pure Organic and Peroxide-Free Redox Initiating Systems for Polymerization in Mild Conditions

**DOI:** 10.3390/polym13020301

**Published:** 2021-01-19

**Authors:** Ahmad Arar, Lilian Wisson, Jacques Lalevée

**Affiliations:** Institut de Science des Matériaux de Mulhouse, IS2M, Université de Haute Alsace (UMR 7361), 15 Rue Jean Starcky, CEDEX 68057 Mulhouse, France; ahmad.arar@uha.fr (A.A.); lilian.wisson@uha.fr (L.W.)

**Keywords:** redox initiators, iodonium salt, gel time, polymerization in mild conditions

## Abstract

Redox initiating systems (RISs) are highly worthwhile for polymerization in mild conditions (at room temperature—RT) without external thermal or light activation. With high performance redox initiating systems RIS, the free radical polymerization FRP can even be carried out under air and without inhibitors/stabilizers removal from the monomers/resins. However, efficient RISs are still based on peroxides or metal complexes. In this work, a pure organic and peroxide-free RIS is presented based on the interaction of a well-selected triarylamine derivative (T4epa) with iodonium salt used as reducing and oxidizing agents, respectively. The redox polymerization (Redox FRP) was followed through pyrometry and thermal imaging experiments. Remarkably, a full control of the work time as well as a high reactivity is observed for mild conditions.

## 1. Introduction

For the production of polymers, free radical polymerization (FRP) is a widespread used process [1]. In this context, thermal or photochemical initiations are mostly used but two-component redox initiation for redox FRP is also a highly robust technique used since the second half of the 20th century [2,3]. Compared to thermal and photo polymerization, redox FRP can be carried out in mild conditions, i.e., without energy consumption as the reaction can be performed out at room temperature without any activation (light or heat). The basic principle of redox FRP is quite straightforward: a redox initiating system (RIS) is dissolved separately in two components (often called cartridges). Then, the mixing of the two components (one containing the oxidizing agent and the second one containing the reducing agent) leads to initiating free radical formation through a redox reaction.

Currently, the benchmark RIS is based on the dibenzoyl peroxide (BPO)/tertiary aromatic amine (e.g., trimethylaniline—TMA) couple. This well-established system is already used in many applications for the preparation of biomedical materials, emulsion polymerization, the curing of composites or adhesives, etc. [4,5,6,7,8,9].

Albeit this benchmark BPO/amine system was the cornerstone of the redox FRP, it is now necessary to replace it due to severe handling/toxicity/hazard issues. Indeed, BPO is not stable (explosive risk associated with peroxides handling, pressing regulations on peroxides, etc.) [10,11] and classical peroxides cannot be stored in monomers leading to highly disymmetrical cartridges (one with the reducing agent in the monomer and a second one for BPO alone). However, under mild conditions (room temperature, under air, no stabilizer removal), alternative systems are not highly efficient [12,13,14,15,16,17,18,19]. This is the reason why we have proposed recently several RIS BPO-free for redox FRP [20,21,22,23,24,25,26].

However, many of the reported systems still involved the presence of metal complexes (Mn, Cu, or V) [20,21,22,23,24,25,26] due to their favorable redox potentials. Therefore, in the present paper, a pure organic and BPO-free approach preventing the use of both peroxides and metal complexes is provided. Specifically, iodonium salts were used as oxidizing agents to generate aryl radicals through their reduction by a carefully selected reducing agent (Tris [4-(diethylamino)phenyl]amine—T4epa) in r1 with ED the electron donor:

ED + Ar_2_I^+^ → ED^●+^ + Ar^●^ + Ar-I (r1)

We have characterized the redox FRP of a benchmark methacrylate monomer blends using different techniques (optical pyrometry and thermal imaging experiments). The workability of the resin through its gel time (GT) is also a crucial parameter: (i) too short gel times does not allow the control the shape of the polymer, i.e., a too fast polymerization occurs; or (ii) long gel time are often associated with low polymerization rates, partial curing, and tacky surfaces. Obviously, the ideal gel time depends on the final application (composites, dentistry, chemical anchors, etc.) but an excellent polymerization must be always obtained in mild conditions (under air, or in presence of stabilizers). In this work, a precise control of the gel time is provided with the new proposed RIS through the contents of redox agents or the use of an additional salts.

## 2. Materials and Methods

### 2.1. Chemical Compounds

**Scheme 1:**
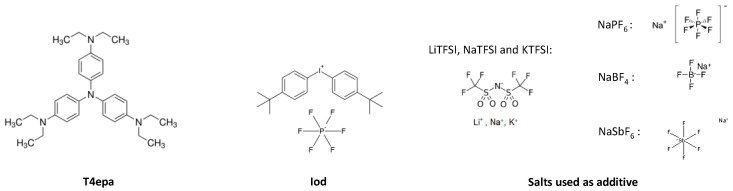
T4epa and iodonium salt (Iod) used in the proposed Redox Initiating System and the salts used as additive in the Redox initiating systems (RISs).

**Scheme 2:**
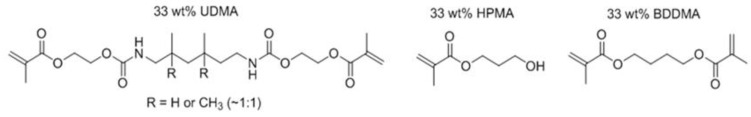
Benchmark methacrylate monomer blend (noted BM for Benchmark Monomers).

All chemicals were purchased with high purity and used as received (Scheme 1). Tris [4-(diethylamino)phenyl]amine (T4epa) and Bis-(4-t-butylphenyl)-Iodonium hexafluorophosphate (Iod) were purchased from Merck-Sigma Aldrich (St. Quentin Fallavier, France) and Lambson Ltd. (Wetherby, UK), respectively. The different salts (NaTFSI, LiTFSI, KTFSI, NaBF_4_, NaPF_6_ and NaSbF_6_; Scheme 1) were ordered from Merck-Sigma Aldrich (St. Quentin Fallavier, France). The efficiency of the different RISs was checked in a benchmark methacrylate monomer blend (noted BM) having an adapted viscosity (0.053 Pa·s) and containing 33.3% UDMA (urethane dimethacrylate), 33.3% HPMA (hydroxypropyl methacrylate), and 33.3% BDDMA (butanediol dimethacrylate) (Scheme 2).

### 2.2. Two Cartridges System Used for Redox FRP Experiments

All redox formulations were prepared in bulk in two separate cartridges: a first cartridge containing T4epa and the other one containing the iodonium salt (Iod). A 1:1 Sulzer mixpac mixer was used to mix the two cartridges at the beginning of each polymerization experiment. As all polymerization experiments were performed at room temperature (~20 to 25 °C) (excepted specifically indicated) and under air, an oxygen inhibition can be expected, particularly for the surface in direct contact with air [20,21,22,23,24,25,26]. For all experiments, the monomers were used without stabilizer (4-methoxyphenol) removal.

### 2.3. Redox FRP in Bulk Followed by Optical Pyrometry

Optical pyrometry was used to follow polymerization reactions: temperature vs. time profiles were followed using an Omega OS552-V1−6 infrared thermometer (Omega Engineering, Inc., Stamford, Paris, France, CT) having a sensitivity of ±1 °C for 2 g samples (thickness ∼ 4 mm) [20,21,22,23,24,25,26]. The redox initiator contents will be given in weight with respect to the monomer in the final blend (wt%). The optical pyrometry is used to check the gel time, i.e., the time required from a two-component mixing to go from fluid/viscous state to gel/solid one.

### 2.4. Redox Polymerization in Bulk Followed by Thermal Imaging Experiments

The temperature reached by the samples has been recorded during the reaction time thanks to an infrared thermal imaging camera (Fluke TiX500, Berlin, Germany) [27].

## 3. Results

### 3.1. The T4epa/Iod RIS Charactaerized by Optical Pyrometry and Thermal Imaging Experiments

**Figure 1:**
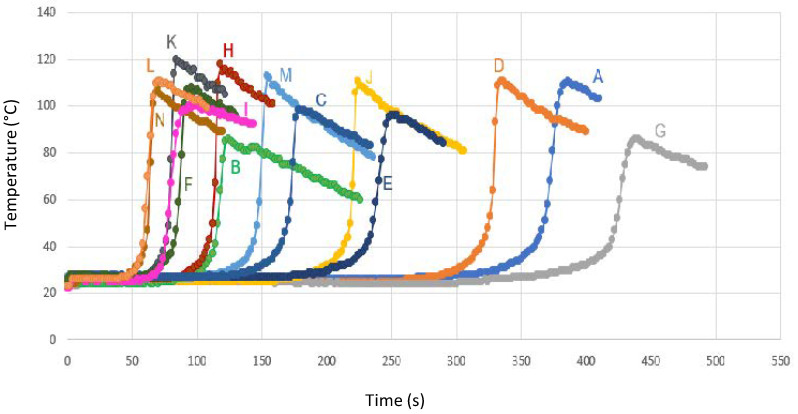
Optical pyrometric measurements (temperature vs. time after mixing; 4 mm thick sample) under air for the BM resin for Redox free radical polymerization (FRP) using different RISs: (**A**) T4epa/Iod (0.5%/1% *w/w*); (**B**) T4epa/Iod (1%/2% *w/w*); (**C**) T4epa/Iod (2%/0.5% *w/w*); (**D**) T4epa/Iod (0.5%/1.5% *w/w*); (**E**) T4epa/Iod (1.5%/0.5% *w/w*); (**F**) T4epa/Iod (2%/1% *w/w*); (**G**) T4epa/Iod (1%/0.5% *w/w*); (**H**) T4epa/Iod (1.5%/1% *w/w*); (**I**) T4epa/Iod (2%/1.5% *w/w*); (**J**) T4epa/Iod (1%/1% *w/w*); (**K**) T4epa/Iod (1.5%/1.5% *w/w*); (L) T4epa/Iod (2%/2% *w/w*); (**M**) T4epa/Iod (1%/1.5% *w/w*); (**N**) T4epa/Iod (1.5%/2% *w/w*).

In this section, the T4epa/Iod RIS is used for the polymerization of the BM resin (Scheme 2) using the 1/1 mixing of the two cartridges, i.e., one containing T4epa in BM and the other one Iod also in BM. The Redox FRP is followed by optical pyrometry that recorded the temperature vs. time after mixing. The FRP process being exothermic, an increase of temperature directly reveals the occurrence of the polymerization reaction. The Gel Time (GT) is defined here as the time where the maximal slope of the temperature is observed (corresponding to the time for the maximal polymerization rate). Remarkably, in Figure 1, T4epa/Iod RIS is found highly efficient to initiate the polymerization of BM in mild conditions (under air, at RT and without stabilizers removal) with GTs ranging from 50 s to 450 s depending on the concentrations of the redox active compounds, i.e., it is worth noting that the GT can be finely controlled by their respective contents. Interestingly, the maximal temperature reached is similar to those obtained for dibenzoylperoxide/trimethylaniline (1%/1%) benchmark system (100 °C and GT = 110 s) suggesting that the new proposed RIS system is competitive in term of redox initiation ability to well-established BPO/amine references. In all case, tack-free polymers were obtained with the proposed systems vs. tacky surfaces for BPO/trimethylaniline highlighting a better ability of the T4epa/Iod RIS to overcome oxygen inhibition vs. BPO/amine reference.

**Figure 2:**
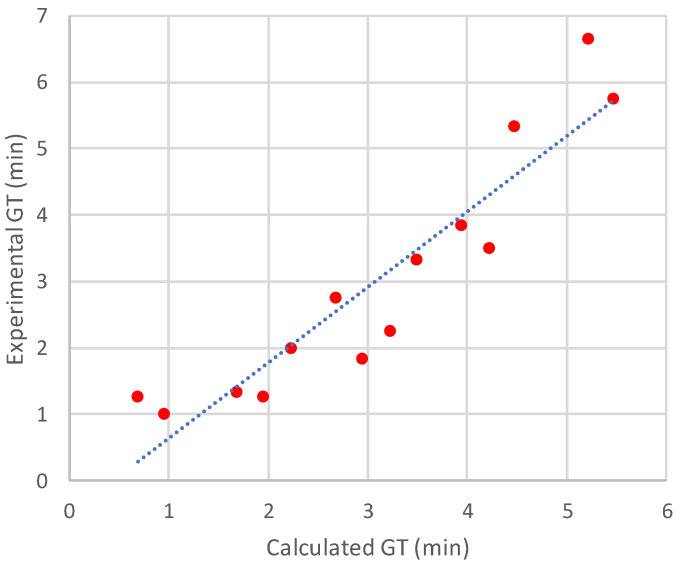
Correlation between the experimental gel time (GT) and the calculated GT using the equation: GT = 8.7 − 2.5 [T4epa] − 2 [Iod].

A multiple regression analysis was carried out to find the relationship between GT and the respective contents of T4epa and Iod (Figure 2). Interestingly, the following equation (Equation (1)) is found for weight contents of Iod andT4epa in the range 0.5–2% (*w/w*):

GT = 8.7 − 2.5 [T4epa] − 2 [Iod] (r^2^ = 0.9) (1)

Equation (1) clearly suggests that an increase of Iod or T4epa content decreases GT. Markedly, rather similar coefficients (2.5 vs. 2) suggest that the content of both compounds have a rather similar effect on GT suggesting a stoichiometric reaction. Such a relationship (Equation (1)) can also be highly worthwhile to control the work time in different applications.

**Figure 3:**
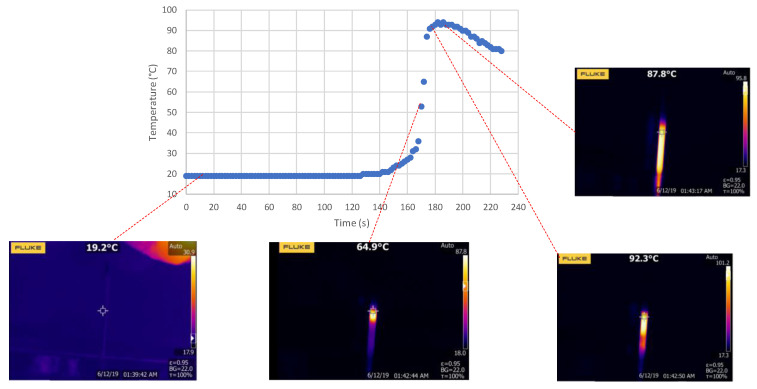
Optical pyrometric measurements (temperature vs. time after mixing; 4 mm thick sample) under air for the BM resin for Redox FRP using the T4epa/Iod (2%/0.6% *w/w*) RIS and the associated thermal imaging pictures at different times.

To better understand the localization of the Redox FRP process in time and in space, thermal imaging experiments in a narrow chemical pill box were realized under air (Figure 3). Remarkably, it is found that the polymerization starts at the top air interface (t = 170 s) before a frontal polymerization occurs from the top to the bottom (see for t = 180s and t = 190 s). This suggests that the oxygen positively participates to the redox initiation in agreement with the ability of the proposed T4epa/Iod RIS to overcome the oxygen inhibition vs. the classical BPO/amine systems that are usually highly inhibited.

### 3.2. Salt Additive Effects in T4epa/Iod RISs

**Figure 4:**
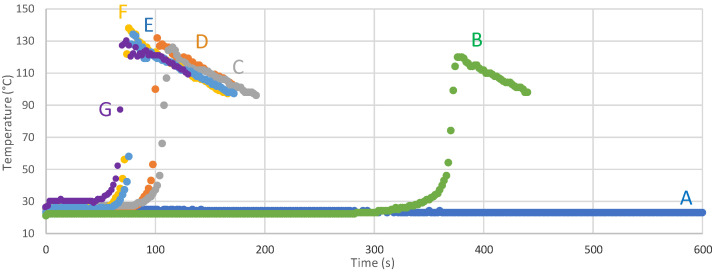
Optical pyrometric measurements (temperature vs. time after mixing; 4 mm thick sample) under air for the BM resin for Redox FRP using different RISs based on T4epa/Iod-Cl (1%/2% *w/w*) in presence of different salts: (**A**) without additional salt; (**B**) NaBF_4_ (1% *w/w*); (**C**) NaSbF_6_ (1% *w/w*); (**D**) LiTFSI (1% *w/w*); (**E**) NaTFSI (1% *w/w*); (**F**) NaPF_6_ (1% *w/w*); (**G**) KTFSI (1% *w/w*).

In this part, an iodonium salt bearing a Chloride anion (noted Iod-Cl) is used instead the iodonium salt of the previous section (noted Iod) based on hexafluorophosphate anion to probe the counter anion effect on the RIS reactivity. Interestingly, for the Iod-Cl, no polymerization occurs in presence of T4epa (Figure 4, curve A) showing that the iodonium bearing PF_6_^-^ is much more reactive. This is related to the much better solubility of Iod vs. Iod-Cl that exhibits a poor solubility in (meth)acrylates. To overcome this latter issue, an in-situ counter anion exchange is investigated by addition of different salts (with different counter anions) in the Iod-Cl cartridge. Markedly, this approach leads to excellent Redox FRP (Figure 4, curves B–G) and the GT can be controlled by the selection of the salt used i.e., GT ~ 370 s with NaBF_4_ and GT ~ 70 s for NaPF_6_. The GT decreases in the series: NaBF_4_ > NaSbF_6_ > LiTFSI > NaTFSI > NaPF_6_ > KTFSI.

**Figure 5:**
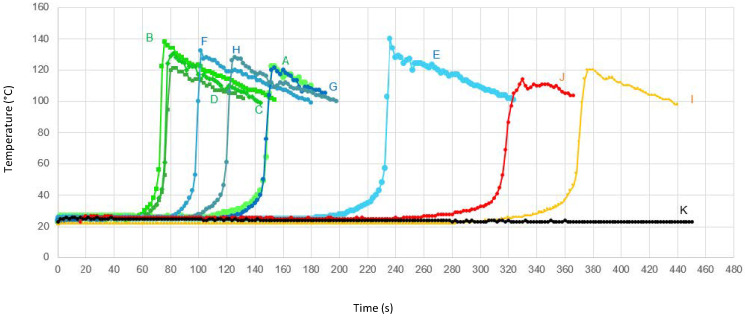
Optical pyrometric measurements (temperature vs. time after mixing; 4 mm thick sample) under air for the BM resin for Redox FRP using different RISs based on T4epa/Iod-Cl (1%/2% *w/w*) in presence of different salts: (**A**) NaPF_6_ (0.5% *w/w*); (**B**) NaPF_6_ (2% *w/w*); (**C**) NaPF_6_ (1% *w/w*); (**D**) NaPF_6_ (1.5% *w/w*); (**E**) LiTFSI (0.63% *w/w*); (**F**) LiTFSI (2% *w/w*); (**G**) LiTFSI (1% *w/w*); (**H**) LiTFSI (1.5% *w/w*); (**I**) NaBF_4_ (1% *w/w*); (**J**) NaBF_4_ (3% *w/w*); (**K**) without additional salt.

The effect of the additional salt concentration was investigated in Figure 5. An increase of the salt concentration leads to a decrease of GT, i.e., GT ~ 150 s for NaPF_6_ (0.5% *w/w*) (Figure 5, curve A) vs. GT ~ 70 s for NaPF_6_ (2% *w/w*) (Figure 5, curve B). The same effect is observed for LiTFSI (Figure 5, curves E–H) and NaBF_4_ (Figure 5, curves I–J). Therefore, the additional salt content is another elegant way to control the work time.

### 3.3. Inhibitor Effects in T4epa/Iod RISs

**Figure 6:**
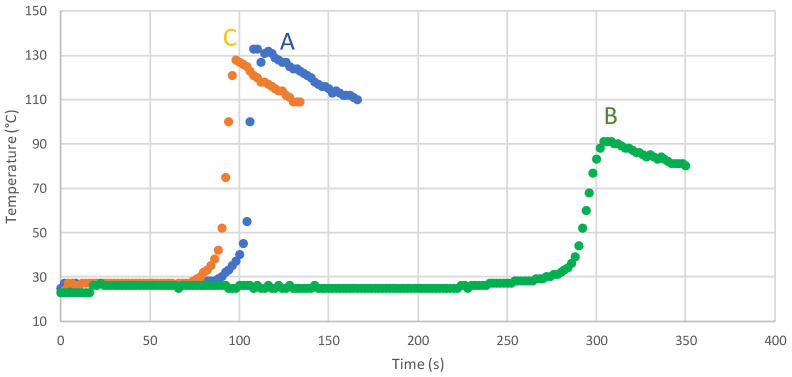
Optical pyrometric measurements (temperature vs. time after mixing; 4 mm thick sample) under air for the BM resin for Redox FRP using a T4epa/Iod (1.5%/1% *w/w*) RIS in presence of different inhibitors: (**A**) without inhibitor; (**B**) 4-Hydroxy-TEMPO (0.05% *w/w*); (**C**) 4-methoxyphenol (0.05% *w/w*).

Additional inhibitors are often used in practical applications to improve the stability of the cartridge and/or to control the gel time. Therefore, the effect of additional inhibitor (4-Hydroxy-TEMPO and 4-methoxyphenol) is investigated in Figure 6. In presence of 4-Hydroxy-TEMPO (0.05% *w/w*), GT increases (Figure 6, curve A vs. curve B) but interestingly, 4-methoxyphenol does not lead to higher GT. Therefore, we can conclude that Hydroxy-TEMPO can be recommended to adjust the GT if necessary while 4-methoxyphenol can be used to improve the stability of the system upon storage without important modification of GT.

### 3.4. Stability in Accelerated Aging Experiments

**Figure 7:**
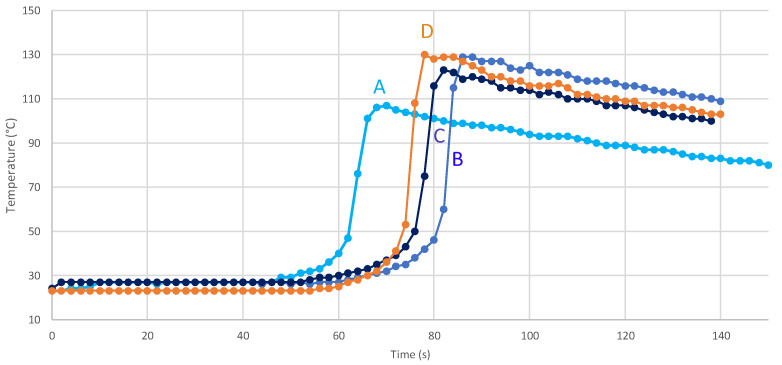
Optical pyrometric measurements (temperature vs. time after mixing; 4 mm thick sample) under air for the BM resin for Redox FRP using a T4epa/Iod (1.5%/1.5% *w/w*) RIS for different storage time: (**A**) fresh cartridges; (**B**) aging of the two cartridges for 7 days @50 °C; (**C**) aging of the two cartridges for 14 days @50 °C; (**D**) aging of the two cartridges for 28 days @RT.

For a deeper characterization of the new proposed T4epa/Iod RIS, the stability of both cartridges in aging experiments has been investigated (Figure 7). A good stability is found both for aging @50 °C (Figure 7, curves B and C) or at RT (Figure 7, curve D) with a low effect on the GT (change < 30 s compared to the fresh cartridges—Figure 7 curve A). keeping in mind the very poor stability of peroxides (particularly BPO) that cannot be stored in monomers, the good stability of Iod and T4epa in BM is a real breakthrough for the Redox FRP field.

### 3.5. Reactivity at Low Temperatures

**Figure 8:**
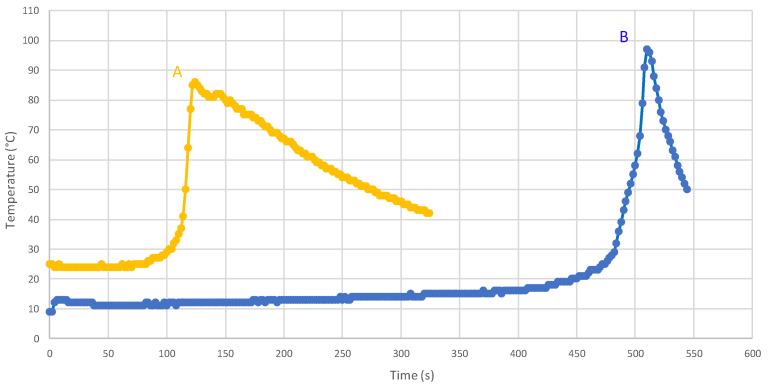
Optical pyrometric measurements (temperature vs. time after mixing; 4 mm thick sample) under air for the BM resin for Redox FRP using a T4epa/Iod (1.5%/1% *w/w*) RIS at different temperatures: (**A**) @RT (23 °C); (**B**) @10 °C.

Finally, Redox FRP can also suffer from lower reactivity at low temperatures. To check the high reactivity of the T4epa/Iod RIS, the polymerizations @25 °C and 10 °C are compared in Figure 8 (curve A vs. curve B). Obviously, a higher GT is obtained @10 °C (~500 s vs. ~120 s) but markedly, without decreasing the exothermicity. Therefore, this result also highlights the robustness of the proposed RIS with polymerization processes that remain possible even at 10 °C.

## 4. Discussion

**Scheme 3:**
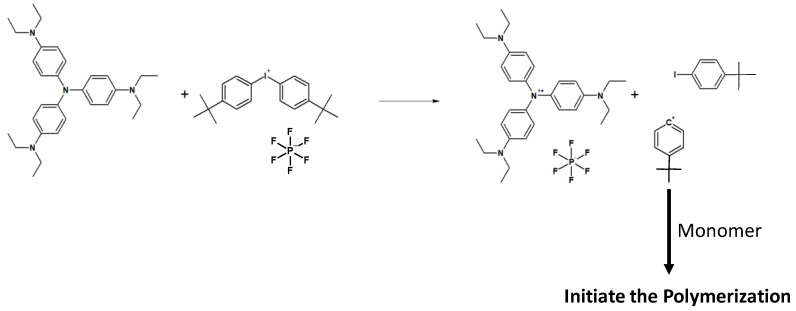
Expected chemical mechanisms.

From all the results obtained above, it clearly appears that the T4epa/Iod RIS is very efficient in mild conditions (under air, without inhibitor removal, and even at low temperature). Taking into account the electron donor properties of aromatic amines and the electron acceptor properties of the iodonium salts, the expected redox reaction is depicted in Scheme 3. The reduction of iodonium salt leads to the formation of highly reactive aryl radials that correspond to highly efficient structures for the addition onto (meth)acrylates with very high addition rate constants (k ~ 10^8^ M^−1^ s^−1^) [28].

It was shown above (see the thermal imaging experiments) that O_2_ can also participate to the chemical mechanisms probably through the consumption of the stabilizer (Ar-OH) in r2–r3 allowing the polymerization to start in contact with air [28].

R^●^ + O_2_ → ROO^●^ (r2)

ROO^●^ + Ar-OH → ROOH + ArO^●^ (r3)

**Scheme 4:**
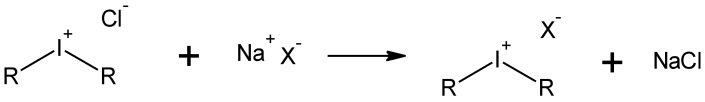
Expected anion exchange to increase the reactivity of iodonium salts in the T4epa/Iod RISs.

Using Iod-Cl in presence of additional salts (e.g., NaX), an in-situ counter anion exchange is expected according to Scheme 4 leading to a more soluble and/or more electron acceptor iodonium salt than the starting Iod-Cl. This latter approach is useful to control the gel time.

## 5. Conclusions

In this work, a pure organic and peroxide-free RIS is presented for polymerization in mild conditions. This latter system is characterized by a bulk polymerization efficiency comparable to the benchmark BPO/TMA system but with the advantage of better surface curing under air. Markedly, a precise control of the gel time is possible through an appropriate content of redox agents or the use of an additional salt. The proposed RIS is highly robust and can be used for polymerization at low temperatures. Other pure and safe organic RIS will be presented in forthcoming works for polymerization. More particularly, peroxide-free systems will be highly welcome.

## Data Availability

The data presented in this study are available on request from the corresponding author.

## References

[1] Braun D. (2010). Origins and Development of Initiation of Free Radical Polymerization Processes. Int. J. Polym. Sci..

[2] Sarac A.S. (1999). Redox Polymerization. Prog. Polym. Sci..

[3] Misra G.S., Bajpai U.D.N. (1982). Redox Polymerization. Prog. Polym. Sci..

[4] Kwon T.-Y., Bagheri R., Kim Y.K., Kim K.-H., Burrow M.F. (2012). Cure Mechanisms in Materials for Use in Esthetic Dentistry. J. Investig. Clin. Dent..

[5] Sideridou I.D., Achilias D.S., Kostidou N.C. (2008). Copolymerization Kinetics of Dental Dimethacrylate Resins Initiated by a Benzoyl Peroxide/Amine Redox System. J. Appl. Polym. Sci..

[6] Sideridou I.D., Achilias D.S., Karava O. (2006). Reactivity of Benzoyl Peroxide/Amine System as an Initiator for the Free Radical Polymerization of Dental and Orthopedic Dimethacrylate Monomers: Effect of the Amine and Monomer Chemical Structure. Macromolecules.

[7] Han D., Meng Z., Wu D., Zhang C., Zhu H. (2011). Thermal Properties of Carbon Black Aqueous Nanofluids for Solar Absorption. Nanoscale Res. Lett..

[8] Achilias D.S., Sideridou I. (2002). Study of the Effect of Two BPO/Amine Initiation Systems on the Free-Radical Polymerization of MMA Used in Dental Resins and Bone Cements. J. Macromol. Sci. Pure Appl. Chem..

[9] Wilson G.O., Henderson J.W., Caruso M.M., Blaiszik B.J., McIntire P.J., Sottos N.R., White S.R., Moore J.S. (2010). Evaluation of Peroxide Initiators for Radical Polymerization-Based Self-Healing Applications. J. Polym. Sci. Part A Polym. Chem..

[10] Sun J., Li Y., Hasegawa K. (2001). A Study of Self-Accelerating Decomposition Temperature (SADT) Using Reaction Calorimetry. J. Loss Prev. Process Ind..

[11] Dunnick J.K., Brix A., Sanders J.M., Travlos G.S. (2014). N,N-Dimethyl-p-Toluidine, a Component in Dental Materials, Causes Hematologic Toxic and Carcinogenic Responses in Rodent Model Systems. Toxicol. Pathol..

[12] Pattnaik S., Roy A.K., Baral N., Nayak P.L. (1979). Aqueous Polymerization of Methyl Methacrylate Initiated by Potassium Peroxydisulfate-Ascorbic Acid Redox System. J. Macromol. Sci. Part A Chem..

[13] Sun X.-L., Sun H.-H., Wang Y.-A. (1998). Polyacrylamide Gel Polymerization: Ascorbic Acid-Ferrous Sulfate-Ammonium Persulfate Initiator for Acid System. Sheng Wu Hua Xue Yu Sheng Wu Wu Li Xue Bao Acta Biochim. Biophys. Sin..

[14] Nayak P.L., Samal R.K., Nayak M.C. (1978). Aqueous Polymerization of Acrylonitrile Initiated by the Mn3+/Citric Acid Redox System. Eur. Polym. J..

[15] Caille J.-R., Debuigne A., Jérôme R. (2005). Quinone Transfer Radical Polymerization (QTRP) of Styrene: Catalysis by Different Metal Complexes. J. Polym. Sci. Part A Polym. Chem..

[16] Van Gorkum R., Bouwman E., Reedijk J. (2004). Fast Autoxidation of Ethyl Linoleate Catalyzed by [Mn (Acac) 3] and Bipyridine: A Possible Drying Catalyst for Alkyd Paints. Inorg. Chem..

[17] Bouwman E., Van Gorkum R. (2007). A Study of New Manganese Complexes as Potential Driers for Alkyd Paints. J. Coat. Technol. Res..

[18] Lavrov N. (1994). Copolymerization of N-Vinylsuccinimide with Vinyl-Acetate in Aqueous-Solution, Initiated by the Manganese Tris(acetylacetonate)-Acetic Acid System. Russ. J. Appl. Chem..

[19] Lavrov N.A. (1995). Kinetic Features of Polymerization of 2-Hydroxyethyl Methacrylate, Initiated by the System Manganese Tris(acetylacetonate)-Acetic Acid. Russ. J. Appl. Chem..

[20] Garra P., Morlet-Savary F., Graff B., Dumur F., Monnier V., Dietlin C., Gigmes D., Fouassier J.-P., Lalevee J. (2018). Metal Acetylacetonate-Bidentate Ligand Interaction (MABLI) as Highly Efficient Free Radical Generating Systems for Polymer Synthesis. Polym. Chem..

[21] Garra P., Dumur F., Morlet-Savary F., Dietlin C., Fouassier J.P., Lalevee J. (2016). A New Highly Efficient Amine-Free and Peroxide-Free Redox System for Free Radical Polymerization under Air with Possible Light Activation. Macromolecules.

[22] Garra P., Dumur F., Morlet-Savary F., Dietlin C., Gigmes D., Fouassier J.P., Lalevee J. (2017). Mechanosynthesis of a Copper Complex for Redox Initiating Systems with a Unique near Infrared Light Activation. J. Polym. Sci. Part A Polym. Chem..

[23] Garra P., Dumur F., Gigmes D., Nechab M., Morlet-Savary F., Dietlin C., Gree S., Fouassier J.P., Lalevee J. (2018). Metal Acetylacetonate–Bidentate Ligand Interaction (MABLI) (Photo)activated Polymerization: Toward High Performance Amine-Free, Peroxide-Free Redox Radical (Photo)initiating Systems. Macromolecules.

[24] Garra P., Dumur F., Nechab M., Morlet-Savary F., Dietlin C., Graff B., Gigmes D., Fouassier J.-P., Lalevee J. (2018). Stable Copper Acetylacetonate-Based Oxidizing Agents in Redox (NIR Photoactivated) Polymerization: An Opportunity for the One Pot Grafting from Approach and an Example on a 3D Printed Object. Polym. Chem..

[25] Arar A., Mokbel H., Dumur F., Lalevee J. (2020). High Performance Redox Initiating Systems Based on the Interaction of Silane with Metal Complexes: A Unique Platform for the Preparation of Composites. Molecules.

[26] Arar A., Mousawi A.A., Boyadjian C., Garra P., Fouassier J.P., Lalevee J.P. (2020). Diphenylsilane-Manganese Acetylacetonate Redox Initiating Systems: Toward Amine-Free and Peroxide-Free Systems, Macromol. Chem. Phys..

[27] Garra P., Bonardi A.H., Baralle A., Mousawi A.A., Bonardi F., Dietlin C., Morlet-Savary F., Fouassier J.P., Lalevee J. (2018). Monitoring Photopolymerization Reactions through Thermal Imaging: A Unique Tool for the Real-Time Follow-Up of Thick Samples, 3D Printing, and Composites. J. Polym. Sci. Part A.

[28] Fouassier J.P., Lalevée J. (2012). Photoinitiators for Polymer Synthesis.

